# Four-Year Follow-Up of a Case of Yellow Nail Syndrome With IgM Deficiency

**DOI:** 10.7759/cureus.55545

**Published:** 2024-03-05

**Authors:** Sagar Nagpal, Sindhu C Pokhriyal, Hezborn M Magacha, David Eisenstadt, Adel El-Abbassi

**Affiliations:** 1 Department of Internal Medicine, East Tennessee State University Quillen College of Medicine, Johnson City, USA; 2 Department of Internal Medicine, One Brooklyn Health/Interfaith Medical Center, Brooklyn, USA; 3 Department of Internal Medicine, East Tennessee State University, Johnson City, USA; 4 Division of Pulmonary Critical Care, East Tennessee State University Quillen College of Medicine, Johnson City, USA

**Keywords:** titanium implants, nail discoloration, pleural effusion, lymphedema, yellow nail syndrome

## Abstract

Yellow nail syndrome is a rare condition occurring sporadically, with an extremely low prevalence rate. This syndrome classically presents with a triad of lower extremity edema, yellow nails, and mucosal issues such as pleural effusion and/or chronic sinusitis. Two out of the three features are deemed sufficient to diagnose a person with yellow nail syndrome. We present a rare case of yellow nail syndrome that began with chronic leg swelling and later progressed to the development of an asymptomatic pleural effusion and finally discoloration of nails. In our case, the patient did have a significant recent history of a total knee replacement with a titanium implant. Of note was the chronology of events including leg edema and asymptomatic pleural effusion which were present even before the titanium knee implant. The third feature of the hardening and yellow discoloration of the nails was found to have developed following the knee replacement. Interestingly, on further evaluation, he was found to have IgM deficiency.

## Introduction

Yellow nail syndrome (YNS) is a rare and complex medical condition characterized by three distinct features: yellowish discoloration and thickening of the nails, respiratory manifestations, and lymphatic abnormalities. It is a rare disorder with an incidence of about 1 in 100,000 [1]. The exact cause of this syndrome remains unknown, making it a challenging disorder to diagnose and manage effectively.

YNS predominantly affects adults, particularly those over the age of 50 years. The primary hallmark of YNS is the yellowish discoloration of the nails, which may affect some or all of the fingernails and toenails. Additionally, the nails can become thickened, brittle, and slow-growing. Respiratory symptoms associated with YNS include chronic cough, shortness of breath, and recurrent lung infections. Lymphatic abnormalities such as lymphedema can manifest as swollen limbs or the accumulation of fluid in body cavities [2,3].

While the pathogenesis of YNS is not fully understood, various hypotheses suggest a combination of lymphatic impairment and altered nail matrix function as contributing factors [2]. To date, no specific genetic or hereditary patterns have been identified. Diagnosing YNS involves clinical evaluation, including nail examination, lung function tests, and imaging studies to assess lymphatic flow. Due to its rarity, treatment approaches for YNS are primarily focused on managing symptoms and improving quality of life [2,4]. Therapeutic options may include topical therapies for nail-related symptoms, antibiotics for respiratory infections, and lymphatic drainage techniques to alleviate swelling. Additionally, lifestyle modifications such as avoiding trauma to the nails and maintaining good respiratory hygiene are recommended [2,4].

Research on YNS is limited, and further investigations are required to uncover its precise underlying mechanisms and develop more targeted interventions. Increased awareness among healthcare professionals and collaboration across different specialties are crucial for early recognition, accurate diagnosis, and optimal management of this challenging condition.

## Case presentation

We present the case of a 79-year-old gentleman with a medical history of hypertension, hyperlipidemia, hypothyroidism, and basal cell carcinoma. He had been experiencing chronic swelling in both legs and recurrent sinusitis with post-nasal drip for a year before seeking medical attention. His past history also included lumbar decompressive laminectomy and surgery for a cerebrospinal fluid leak six months before his initial presentation. Subsequently, the patient underwent unilateral total knee replacement with a titanium implant for osteoarthritis almost a year after the initial clinical presentation.

**Figure 1:**
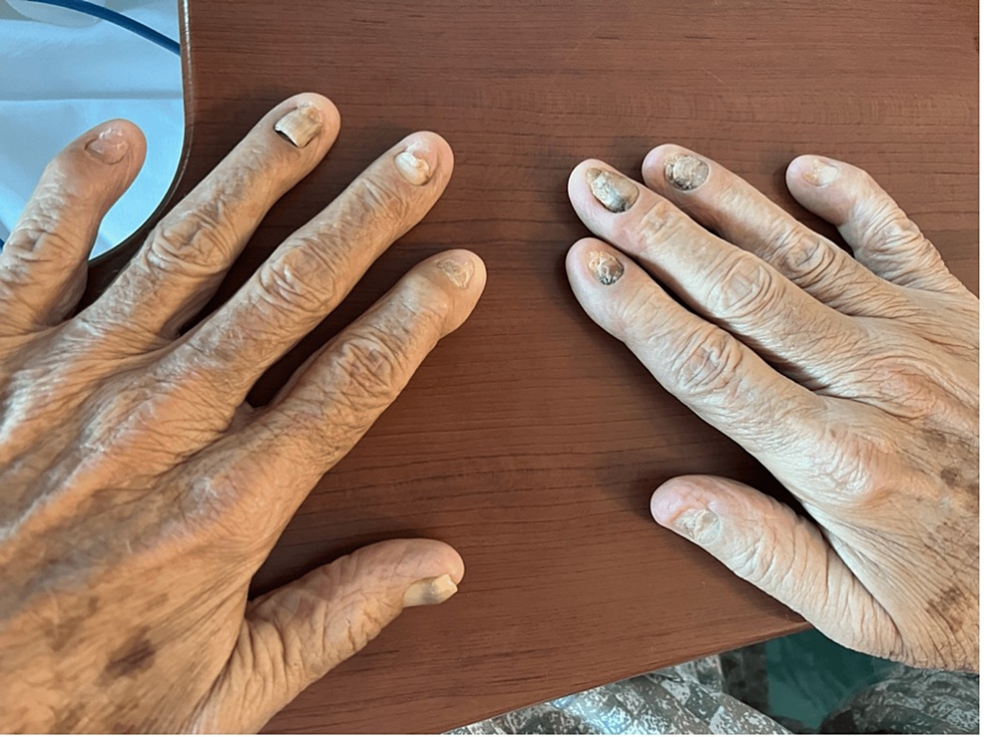
Image of the hands showing yellow and dystrophic nails.

**Figure 2:**
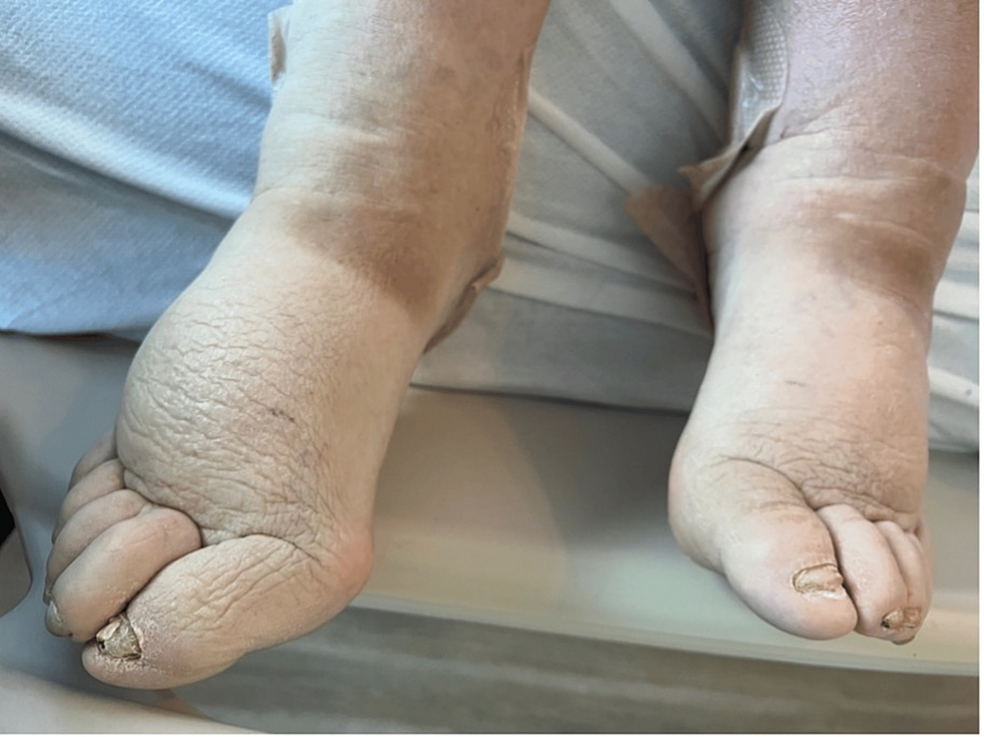
Image showing significant lower extremity edema and nail changes.

**Figure 3:**
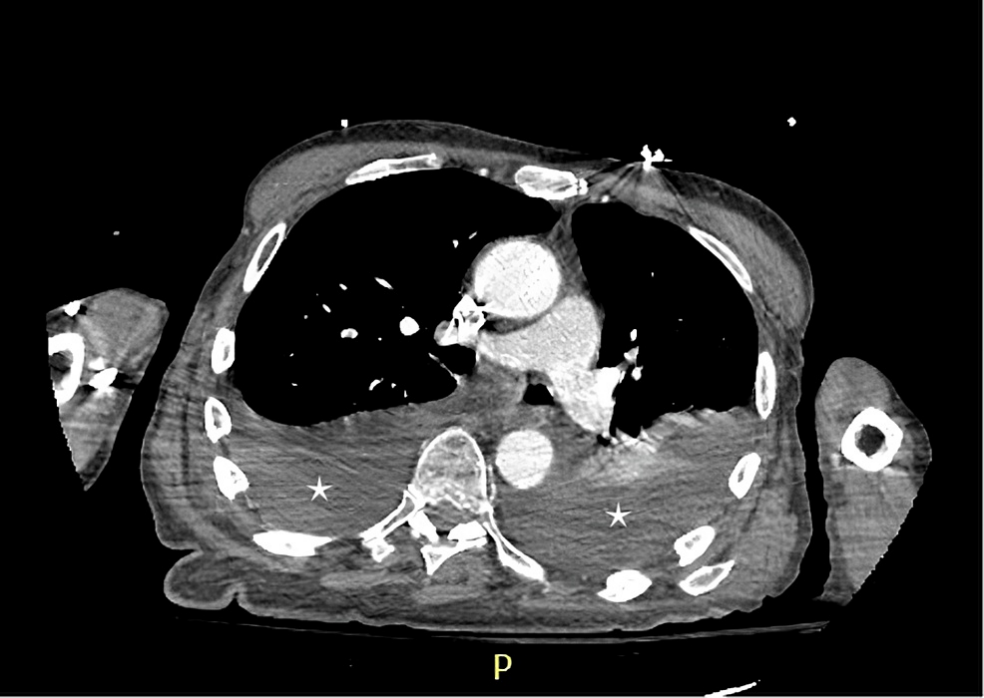
Computed tomography scan of the lungs showing bilateral pleural effusions (marked with *).

Following the knee replacement, the patient developed complaints of slow-growing fragile nails with yellow discoloration. He also reported numbness and tingling in both upper extremities over the past month. Physical examination revealed bilateral non-pitting edema, yellow discoloration of nails in both hands and feet (Figures 1, 2), scattered crackles on auscultation, and decreased air entry in the lower lung fields bilaterally. Venous duplex imaging of the lower extremities ruled out venous thrombosis. Chest X-ray and computed tomography of the chest revealed bilateral pleural effusions (Figure 3).

The combination of pleural effusion, non-pitting edema, and yellow nail discoloration led to the clinical diagnosis of YNS. Due to the reported association of YNS with malignancy, an extensive workup for cancer was conducted, including a whole-body positron emission tomography scan, which yielded negative results. Further investigations for connective tissue disorders revealed a positive antinuclear antibody screen with a homogenous pattern and a titer of 1:160. The rest of the immunological workup was negative. Additionally, an immunological workup identified selective IgM deficiency with an IgM level of 26 mg/dL. Electromyography was performed to assess paresthesia and revealed distal neuropathy in the digits of both upper extremities.

The patient was followed for the next four years, during which he continued to experience disabling bilateral lower extremity edema and persistent asymptomatic small bilateral pleural effusions. Various therapies were attempted for symptomatic relief, including furosemide and vitamin E, but they were found to be ineffective. Over the following years, the patient received massage therapy and wore a compression bandage for leg edema, which eventually improved his symptoms and functionality.

In the fourth year after the initial presentation, the patient’s pleural effusion significantly increased, causing respiratory distress. Drainage was planned, and thoracentesis was performed, removing 1,200 mL of pleural fluid from the left pleural cavity and 1,600 mL from the right side. Fluid analysis indicated an exudative effusion, and cytology revealed the presence of lymphocytes. Sputum and pleural fluid cultures were negative for infections. The patient recovered well following the thoracentesis, and subsequent follow-up appointments showed small-sized bilateral pleural effusions that were still present but asymptomatic.

## Discussion

When Heller et al. first described YNS in 1927, they included only yellow discoloration of nails and lymphedema as the diagnostic criteria [1]. The clinical features of pleural effusion and other respiratory tract mucositis were included in the diagnostic criteria only in 1966 [5]. YNS predominantly affects men over the age of 50 years [6].

Although pleural effusion is the classic respiratory tract finding, other findings such as chronic cough, recurrent pneumonia, recurrent episodes of sinusitis, bronchiectasis, and pulmonary fibrosis are also seen often [7]. Valdés et al. in a review of 66 patients with YNS reported that the pleural effusion was almost always lymphocytic-predominant exudate and was bilateral 70% of the time [1,8]. At the advent, Emerson et al. described the need for all three findings to be present for diagnosing YNS. However, in 1972, Heller et al. first described that two out of three of the clinical features are sufficient to diagnose YNS [9]. The etiopathogenesis of YNS is still not well established. A functional abnormality in lymphatic drainage has often been implicated in the etiology of YNS [10].

YNS is a diagnosis of exclusion, and although the diagnosis is based on the presence of two out of three of the three previously named clinical features, a workup is required to rule out malignancy, infectious etiology, and heart failure [6]. YNS has also been associated with multiple autoimmune disorders such as Hashimoto’s thyroiditis, rheumatoid arthritis, common variable immune deficiency, and Guillain-Barré syndrome. Apart from this, there have been reports of the association of YNS with certain malignancies, suggesting that it might present as a telltale paraneoplastic syndrome [1]. In their recent literature review, Cheslock et al. suggested some investigations for the evaluation of YNS which included echocardiography, chest X-ray, sputum analysis, white blood cell count with differentials, thoracentesis in the presence of pleural effusion, and lymphoscintigraphy to check for lymphatic insufficiency, which is considered a hallmark of YNS [6].

Nail disease in YNS can be managed medically or with surveillance as spontaneous resolution has been seen in some cases. Evidence supporting the use of oral vitamin E to treat nail discoloration is inconclusive [11]. There is insufficient evidence to support the use of antifungals alone or in combination with vitamin E, oral zinc, clarithromycin, and corticosteroid injections [6].

The standard management for pleural effusions is thoracentesis. Octreotide has shown efficacy in managing YNS-related chylous effusions in some cases [12].

Management of lymphedema involves using non-surgical interventions such as compression garments and bandaging in conjunction with hygiene, manual lymph drainage, and exercises [13].

Titanium’s role in the pathogenesis of YNS is unknown and speculative. Cosmetics, sunscreens, medications, candies, and joint implants frequently contain titanium dioxide [14]. Berglund and Calmark proposed that titanium dioxide played a role in the development of YNS. Their study revealed elevated titanium levels in nail clippings from 30 YNS patients while titanium was not detected in the nails of healthy cohorts. After removing their titanium implants, four patients experienced complete recovery [15]. Dos Santos in their research questioned the association of titanium exposure with YNS [16]. Their study on the relationship between titanium pigment autopsied patients with significant titanium exposure found that in some patients even though the titanium pigment was found in several organs including the liver, spleen, lymph nodes, and lungs, it was not accompanied by yellowish discoloration of nails in these patients [16].

Furthermore, a study of five Brazilian patients by de Lima et al. found titanium deposits in samples of the liver, spleen, lungs, lymph nodes, and bone marrow, but there was no change in the pigment or structure of the nails in these patients. Despite the chronic presence of titanium throughout the body, no yellow nail changes were seen in these patients [17]. Additional research is required to establish a direct causal relationship. YNS is a functionally debilitating condition due to the associated respiratory issues as well as lymphedema, which can compromise the activities of daily living [14].

The pathogenesis of YNS is not completely understood. Our patient had a classic triad of non-pitting edema, pleural effusion, and nail changes. As per the current definition of YNS, the patient met the criteria of two out of three features even before the knee replacement surgery. The inclusion of yellow nail discoloration as a mandatory criterion alongside the other two characteristics and its impact on the management of YNS warrants further consideration.

The chronology of events in our case supports the uncertainty in the syndrome that could not be attributed completely to the titanium implant as our patient had symptoms of effusion and non-pitting edema before knee arthroplasty. He developed nail changes after the knee implant. We attribute the onset of the syndrome to lumbar surgery, not as a complication of surgery, but as an idiosyncratic response to surgery. We suggest a relook at the current definition of YNS as well as suggest more research to establish the causal relationship between titanium exposure and YNS.

## Conclusions

Further studies need to be done to establish a causal relationship between titanium and YNS. Considering the chronology of events in our patient, it might well be that patients with mucositis are more prone to developing YNS following titanium implants. We also recommend conducting randomized controlled studies where all patients undergoing titanium implants are closely followed for the development of YNS, given the rampant use of such implants. Additionally, patients with YNS should be evaluated for coexisting immunodeficiency disorders.
